# Remodeling of the Basal Labyrinth of Retinal Pigment Epithelial Cells With Osmotic Challenge, Age, and Disease

**DOI:** 10.1167/iovs.19-26784

**Published:** 2019-06

**Authors:** Matthew J. Hayes, Thomas Burgoyne, Silene T. Wavre-Shapton, Tanya Tolmachova, Miguel C. Seabra, Clare E. Futter

**Affiliations:** 1 University College London, Institute of Ophthalmology, London, United Kingdom; 2 Imperial College London, London, United Kingdom; 3 CEDOC, NOVA Universidade Nova de Lisboa, Lisbon, Portugal

**Keywords:** basal infoldings, retinal pigment epithelium, mitochondria

## Abstract

**Purpose:**

The basal surface of the retinal pigment epithelium (RPE) is folded into a complex basal labyrinth thought to facilitate solute and water transport. We aimed to analyze and define the structural organization of the basal labyrinth of the RPE to enable quantitative analysis of structural changes in age and disease and to better understand the relationship between basal labyrinth structure and efficiency of transepithelial transport.

**Methods:**

Conventional transmission and serial block-face scanning electron microscopy and electron tomography were used to examine the structure of the basal labyrinth in mouse eyes of different ages and genotypes and with and without osmotic shock before fixation.

**Results:**

We identified structurally distinct zones (stacked and ribbon-like) within the RPE basal labyrinth that are largely organelle free and cisternal elements that make contact with the endoplasmic reticulum (ER) and mitochondria. These zones are lost in a hierarchic fashion with age and prematurely in a model of the progressive retinal degenerative disease, choroideremia. Junctional complexes crosslink closely opposed infoldings. Spacing between the basal infoldings was affected by subtle osmotic changes while osmotic shock induced dramatic remodeling of the infoldings.

**Conclusions:**

The basal labyrinth has complex but ordered structural elements that break down with age and in choroideremia. The geometry of these elements and site of contact with ER and mitochondria likely facilitate the ion transport that drives water transport across the basal RPE surface. Changes in structure in response to local osmotic variation may allow transport to be modulated in order to maintain RPE volume.

The retinal pigment epithelium (RPE), Bruch's membrane, and the endothelial cells of the choriocapillaris constitute the blood–retinal barrier across which nutrients, metabolites, solutes, and water are transported.^1^–^3^ The apical and basal surfaces of the RPE exhibit complex plasma-membrane elaborations that increase the effective surface area over which exchange can occur in order to meet the high-metabolic demands of the eye.

Loss of the basal infoldings that form the basal labyrinth of the RPE, or the accumulation of material within these elaborations, correlates with aging,^4^–^7^ smoking,^8^ and has been implicated in retinal degeneration.^9^ Such changes are also a feature of animal models of eye disease,^7^,^10^–^12^ including our previously generated mouse models of choroideremia (CHM), a disease caused by loss of Rab Escort Protein 1 (REP1).^13^ Rab GTPases are regulators of membrane trafficking^14^ and loss of function of REP1 in CHM results in reduced Rab prenylation, a lipid modification necessary for Rab membrane binding and function.^13^ REP2 can partially compensate for loss of REP1 and so CHM is characterized by partial defects in membrane trafficking pathways, the effects of which accumulate over time leading to gradual degeneration of RPE, photoreceptors, and choroid. Cre-Lox–mediated tissue-specific (RPE and melanocyte) knockout of REP1 resulted in premature accumulation of features of aging in the RPE, including a striking disorganization of the basal infoldings.^15^ The space between Bruch's membrane and basal membrane of the RPE is also the site of basal laminar deposit and drusen accumulation; often considered to presage AMD. Loss of REP1 in the mouse RPE led to accumulation of basal laminar deposits, frequently associated with disorganized basal infoldings, but whether this disorganization was causally linked with or the result of basal deposit formation was unclear.^15^

The architecture of the basal infoldings has the potential to profoundly influence transepithelial transport and RPE cell adhesion. Transepithelial transport is tightly regulated to preserve the isotonicity of the neural retina and prevent accumulation of fluid in the subretinal space.^3^ The drive for transepithelial water flux is provided by active ion transport mediated by a variety of ion transporters, including Na^+^-K^+^-2Cl^−^, and Na^+^ and 2HCO_3_^−^ co-transporters on the apical surface and Cl^−^ and HCO_3_^−^ transporters on the basal surface.^16^,^17^ Aquaporins on both surfaces are potential conduits for the water that follows the ion gradients thus established. The geometry of the basal infoldings potentially alters the transepithelial water flow by modifying the shape of the ionic gradient in the vicinity of the basal membrane and so loss of this architecture could affect both RPE cell volume and adhesion.

Examination of published thin-section transmission electron micrographs (TEM) suggests considerable variation in basal infolding organization within the retina and high sensitivity to postmortem changes (in human retina). We set out to perform a three-dimensional (3D) analysis of the basal infolding architecture with the aim of better understanding its geometric organization and to quantitatively characterize changes in architecture during aging and disease. This should facilitate future mechanistic studies aimed at determining how the architecture is established and maintained and how these mechanisms break down in age and disease.

## Methods

### Mice

All animals used in this study were treated humanely in accordance with Home Office guidance rules under project license 70/6176 and 70/7078, adhering to the ARVO Statement for the Use of Animals in Ophthalmic and Vision Research. The conditional knock-out mouse line *Chm^Flox^Tyr-Cre* (Chm), carrying the Cre-recombinase transgene under control of the tyrosinase promoter, was generated previously and genotyping of mice was performed as described.^18^ *Chm^Flox^* (littermates without Tyr-Cre transgene) and *ChmWT* mice were used as controls. Both females and males were used in this study and all strains were of the C57Bl6 background.

### Transmission Electron Microscopy (TEM)

Mouse eyes were fixed and embedded as described.^15^ Ultra-thin sections were stained with lead citrate before imaging in a JEOL 1010 TEM (Welwyn Garden City, UK) with a Gatan Orius SC100B charge-coupled device camera (Gatan Inc, Abingdon, UK). For tomography, 250-nm sections were prepared and stained with fiducial markers for subsequent alignment (10-nm colloidal gold solution). Images were acquired using a FEI T12 Tecnai Spirit electron microscope equipped (Milton Park, UK) with a Morada camera (Olympus SIS, Southend-on-Sea, UK) and iTEM tomography software (Olympus SIS). Tilt series (2 orthogonal axes) were collected from −70° to +70° in 2° increments. The tilt series were aligned and a dual axis tomogram generated and modeled using IMOD.^19^

### High Pressure Freezing/Freeze Substitution

Tissue-punched fresh mouse eyecup was placed into copper carriers (0.4-mm depth) and frozen at high-pressure using a Leica EM PACT (Leica Microsystems, Vienna, Austria). Freeze substitution in the automated Leica AFS2 used the following incubations: (1) 0.1% tannic acid in acetone for 22 hours at −90°C, (2) acetone for 2 hours at −90°C, (3) 2% osmium and 0.2% uranyl acetate in acetone for 28 hours from −90°C to −30°C, (4) acetone for 17 hours from −30°C to room temperature, (5) propylene oxide for 20 minutes at room temperature, and (6) propylene oxide and epon at 1:1 for 1 hour at room temperature. Finally, samples were embedded in epon overnight at 60°C.

### Serial Block-Face Scanning Electron Microscopy (SBF-SEM)

Eyes were fixed in 2% paraformaldehyde/2% glutaraldehyde in 0.1 M sodium cacodylate buffer (pH 7.4) at room temperature for 2 hours, before incubating in the following solutions: 1% aqueous osmium tetroxide and 1.5% potassium ferrocyanide at 4°C for 1 hour at room temperature, 1% aqueous thiocarbohydrazide for 20 minutes at room temperature, and 2% aqueous osmium tetroxide for 30 minutes at room temperature. En bloc staining was performed by incubating the eyes in 1% aqueous uranyl acetate at 4°C overnight followed by Walton's lead aspartate solution for 30 minutes at 60°C. The eyes were dehydrated using increasing concentrations of ethanol and propylene oxide followed by infiltration with 50:50 Durcupan ACM resin:propylene oxide (Sigma-Aldrich, Gillingham, UK) at room temperature overnight, before embedding in Durcupan ACM resin at 60°C overnight. Small blocks cut from the embedded eye were mounted onto aluminum cryo-ultramicrotome pins and sputter-coated with a layer of platinum (Cressington 108; Cressington, Watford, UK). In between sequential sectioning of the block face using a Gatan 3View System (Gatan Inc., Abingdon, UK) images were collected in a Zeiss Sigma VP field emission SEM (Zeiss, Cambridge, UK). The images were aligned using the StackReg plugin (EPFL, Lausanne, Switzerland) in ImageJ software (http://imagej.nih.gov/ij/; provided in the public domain by the National Institutes of Health, Bethesda, MD, USA) and modeling of the basal infoldings was performed using IMOD.^19^

### Quantitative Image Analysis

Basal infolding widths, areas of paracellular space, and cytoplasmic area occupied by different basal infolding zones were outlined by hand and measured using ImageJ.

### Osmotic Stress Experiments

Eyes were placed into Eppendorf tubes containing iso- (140 mM NaCl, 1 mM CalCl_2_, 1 mM MgCl_2_, 5.5 mM glucose, 0.1% BSA, 20 mM HEPES), hyper- (iso- plus 20 mM sucrose), or hypo- (iso-diluted 50% with 1 mM CalCl_2_, 1 mM MgCl_2_, 5.5 mM glucose, 0.1% BSA, 20 mM HEPES) osmotic buffer for 10 minutes at room temperature, and fixed by the addition of an equal volume of 5% paraformaldehyde and 4% glutaraldehyde in 0.1 M sodium cacodylate buffer (pH 7.4) for 2 hours at room temperature.

## Results

### SBF-SEM Allows Three Structurally Distinct Zones of the RPE Basal Infoldings to be Identified

**Figure 1:**
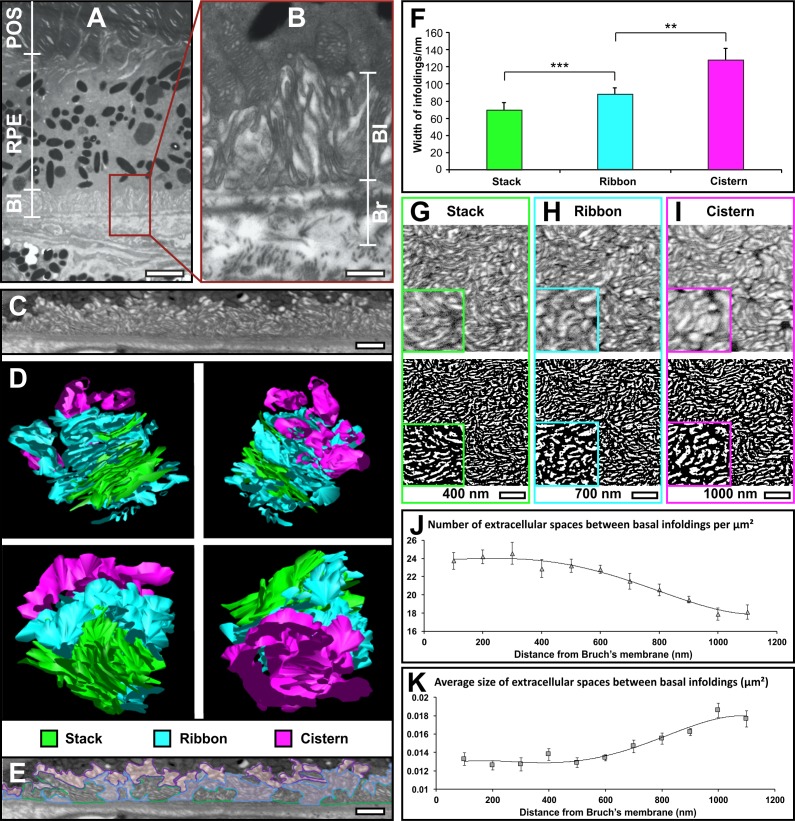
RPE basal infoldings are organized into three distinct structural zones. (A, B) Electron micrograph of 5-month-old mouse RPE exposed to photoreceptor outer segments (POS) apically and Bruch's membrane (Br) basally. What appear to be filopod-like basal infoldings (BI) extend from the cell body to rest against Bruch's membrane as shown in (A). (B) A higher magnification image of the boxed region in (A). (C–E) SBF-SEM data that show basal infoldings have a complex but ordered architecture. (C) A single image from the SBF-SEM image stack that was used to generate the 3D model (D) of the basal infoldings. Within this model three distinct structural zones could be identified, stacks (green), ribbons (blue), and cisterns (pink). By identifying and characterizing these in 3D, stack, ribbons, and cisterns could be identified within single SBF-SEM image slices (E). (F) Measurement of the mean distance between membranes of individual basal infoldings in the different structural zones revealed an increase in width from the stacks to the cisterns (results are means ± SD >200 individual infoldings. Student's t-test **P < 0.05, ***P < 0.01). (G–I) Image slices at 400, 700, and 1000 nm from Bruch's membrane from an SBF-SEM sample sectioned en face (upper panels) and converted to binary format (lower panels) to allow measurement of number (J) and size (K) of extracellular spaces, showing reduced number and increased size moving away from the Bruch's membrane (results are means ± SEM of 4 different areas at each height). Scale bars, (A) 2.5, (B) 0.5, (C, E) 1, and (G–I) 1 μm.

In ultrathin cross sections of chemically fixed 5-month-old mouse eyes, the RPE basal infoldings appear to be elongated filopods that originate from a fairly consistent level of the cell and extend basally to rest their tips on Bruch's membrane (Figs. 1A, 1B).

To better understand the 3D organization of the basal infoldings, we prepared 5-month-old mouse eyes for SBF-SEM (see Supplementary Videos S1 and S2). Single scans (Fig. 1C) revealed structure similar to that revealed by conventional thin-section TEM. Three-dimensional reconstructions of SBF-SEM images showed the structure of the basal infoldings to have a complex but ordered architecture composed of three structurally distinct zones (cisterns, ribbons, and stacks) (Figs. 1D, 1E) that differ in both the width of the basal infoldings themselves (Fig. 1F) and the paracellular space between them (Fig. 1G–K). Examination of single images of a data set obtained by sectioning longitudinally reveals how the zones are arranged in relation to each other and to the cell body and Bruch's membrane (Fig. 1E). Examination of single images at different heights through a data set obtained by sectioning en face (see Supplementary Video S2) allows the number and size of extracellular spaces between the basal infoldings to be accurately measured (Figs. 1G–K). The basal ‘zone', closest to Bruch's membrane, is a series of closely opposed membranous sheets that we term ‘stacks' (green in Fig. 1D). Not all sheets in a stack reach as far as the basal lamina and neither do all stacks, though most do. These stacks are held at a range of orientations but most are approximately perpendicular to Bruch's membrane. In these regions there is a large number of small paracellular spaces between adjacent basal infoldings (Figs. 1G, 1J, 1K), reflecting the dense packing. Adjacent to this zone, closer to the cell body, there are more complex regions we term ‘ribbon-like' (blue in Fig. 1D). In this region the membranous sheets are not stacked and there are larger paracellular spaces (Figs. 1H, 1J, 1K), reflecting the more disordered organization. Closest to the cell body the paracellular space between the basal infoldings opens out into cavernous cisternae (pink in Fig. 1D), such that there are fewer, larger paracellular spaces, than in either stacked or ribbon-like zones (Figs. 1I, 1J, 1K). The cisternal spaces are often intruded into by one or more blunt filopod like protrusions.

### The Proportion of Each Type of Basal Infolding Changes With Age and Prematurely in the *Chm^Flox^Tyr-Cre* Mouse

**Figure 2:**
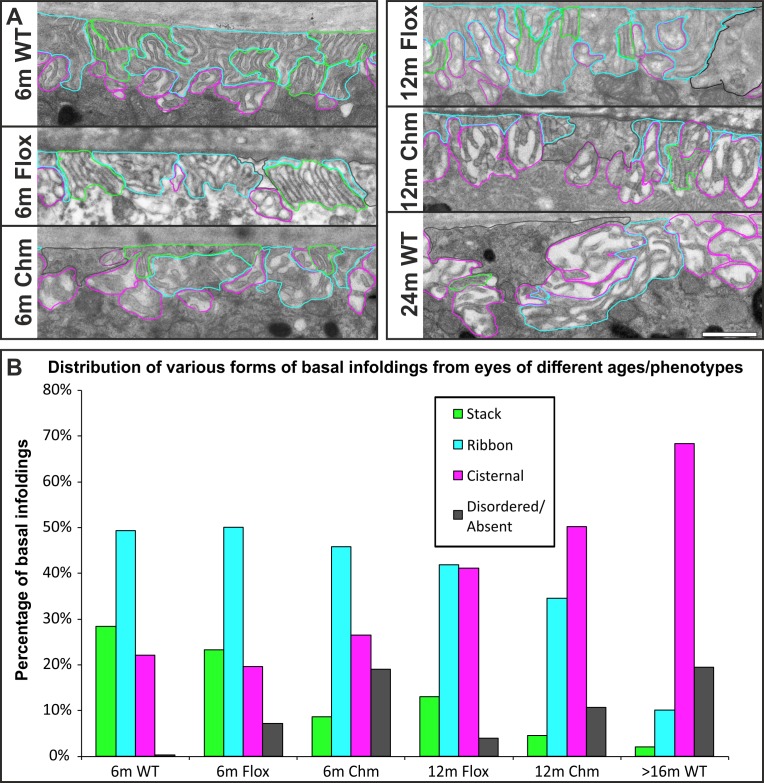
Basal infolding architecture changes with age and in a choroideremia mouse model. (A) The different basal infolding structural zones were identified (stacks in green, ribbon in blue, and cisterns in pink as well as disordered regions or regions without basal infoldings in dark gray) in control ChmWT (WT), Chm^Flox^ (Flox), and the Chm^Flox^Tyr-Cre (Chm) model mice at 6, 12, and 24 months. (B) The distribution of each basal infolding zone within the mouse models examined showed a noticeable reduction in stacks and an increase in cisternal basal infoldings with age and in Chm (compared with controls of the same age). Regions where infoldings were disordered or absent also increased with age and in Chm. Two to four eyes were analyzed for WT and Flox and three to four for Chm. Scale bar, (A) 1 μm.

The 3D information provided by SBF-SEM was necessary to establish the three different structural zones of the RPE basal labyrinth, and this insight enabled us to reinterpret features of basal infoldings seen in conventional TEM. Stacks, ribbons, and cisternae were all identifiable in thin sections (Fig. 2A) allowing us to quantitatively analyze the distribution of the different basal infolding subtypes in mice of different ages and in mice lacking REP1 in the RPE (Chm) (Fig. 2B). In 6-month-old *ChmWT* and *Chm^Flox^* control mice there was an approximately equal proportion of stacked and cisternal zones and a higher proportion of ribbon like zones. In older wild-type mice there was a loss of stacked zones and an increase in cisternal zones and there were also areas where basal infoldings were lost altogether (not present in retinae from 6-month-old wild-type mice). Consistent with loss of REP1 in the RPE-inducing changes associated with aging, 6- and 12-month-old *Chm^Flox^Tyr-Cre* mice exhibited a loss of stacked zones and increase in cisternal zones and zones lacking basal infoldings that was much greater than in age-matched *Chm^Flox^* controls (Fig. 2B).

Supplementary Figure S1 shows examples of data from individual eyes, showing variation in membrane architecture in different regions. In some regions, the membrane architecture is relatively constant but other areas of the same eye sections contain regions (composed of just a few juxtaposed cells) of different architectures.

### Junctions and Bridges Connect Adjacent Basal Infoldings

**Figure 3:**
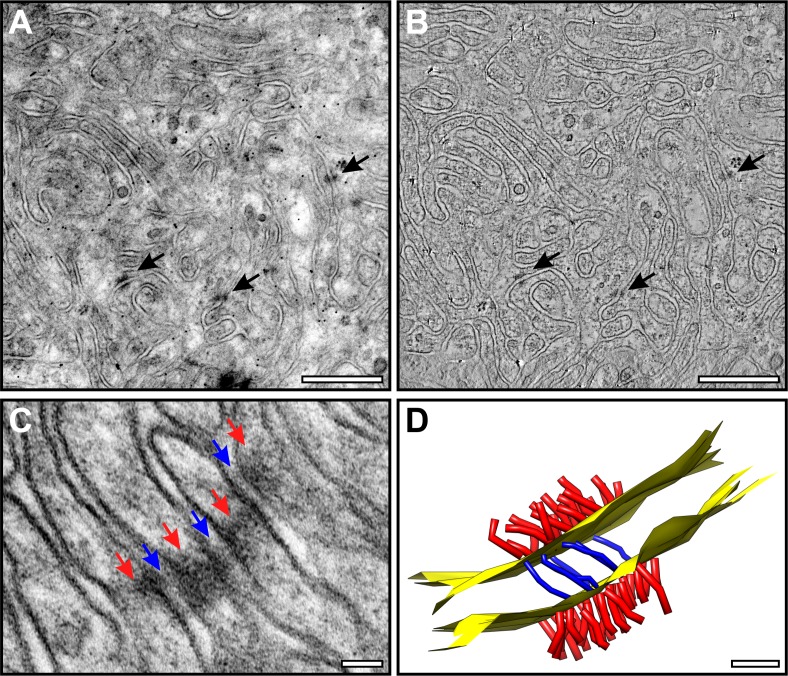
Basal infoldings are linked together by junctional complexes. (A, B) En face sections through the basal infoldings of 5-month-old wild-type mouse retina revealed adherens-like junctions (black arrows) linking adjacent basal infoldings in conventional electron micrographs (A) and a slice from a tomographic reconstruction of the same area (B). (C, D) Junctional filaments can be seen within the basal infoldings ([C] red arrows, [D] red filaments) as well as bridging membranes across the extracellular space ([C] blue arrows, [D] blue filaments) as shown in (C) an electron micrograph image and (D) a model generated from the tomographic reconstruction. Scale bars, (A, B) 500, (C) 50, and (D) 20 nm.

We identified small (50 ± 21 nm), electron-dense junctional complexes that appear to interconnect the membranes of closely apposed basal infoldings. These are most obvious when the basal infoldings are sectioned en face (Figs. 3A, 3B). Electron tomography reveals these electron densities associated with the plasma membrane to be composed of short, densely packed filaments on the cytoplasmic side and a small number of loosely packed filaments extending across the paracellular space (Figs. 3C, 3D).

Measurements from montages of high-magnification images reveal that the junctions occur at intervals of approximately one per 1 μm of plasma membrane and they are found at all ‘depths' of the basal labyrinth. These junctional complexes share morphological features with both desmosomes and adherens junctions, and so we attempted to localize components of these junctions by cryo-immuno EM. Unfortunately, we were unsuccessful due to a combination of factors as follows: (1) low-labeling efficiency of this technique, (2) the technical demands of achieving the right section plane to visualize the junctional complexes by cryo-ultramicrotomy, and (3) the weaker fixation required to maintain antigenicity making the structural preservation of the basal labyrinth less effective than in conventional EM. Overcoming the requirement for cryo-ultramicrotomy, by embedding specimens in London Resin (LR) White that can be sectioned at room temperature, resulted in inadequate tissue preservation and, as a consequence, a lack of both immunogold staining and identifiable junctions. Attempts by pre-embedding labeling, followed by conventional EM, were also unsuccessful, possibly due to inaccessibility of antigen following the very mild permeabilization of the tissue that was necessary to maintain the structural integrity of the basal labyrinth.

### Basal Infoldings Lack Organelles but Cisternal Zones Make Contact With ER and Mitochondria

**Figure 4:**
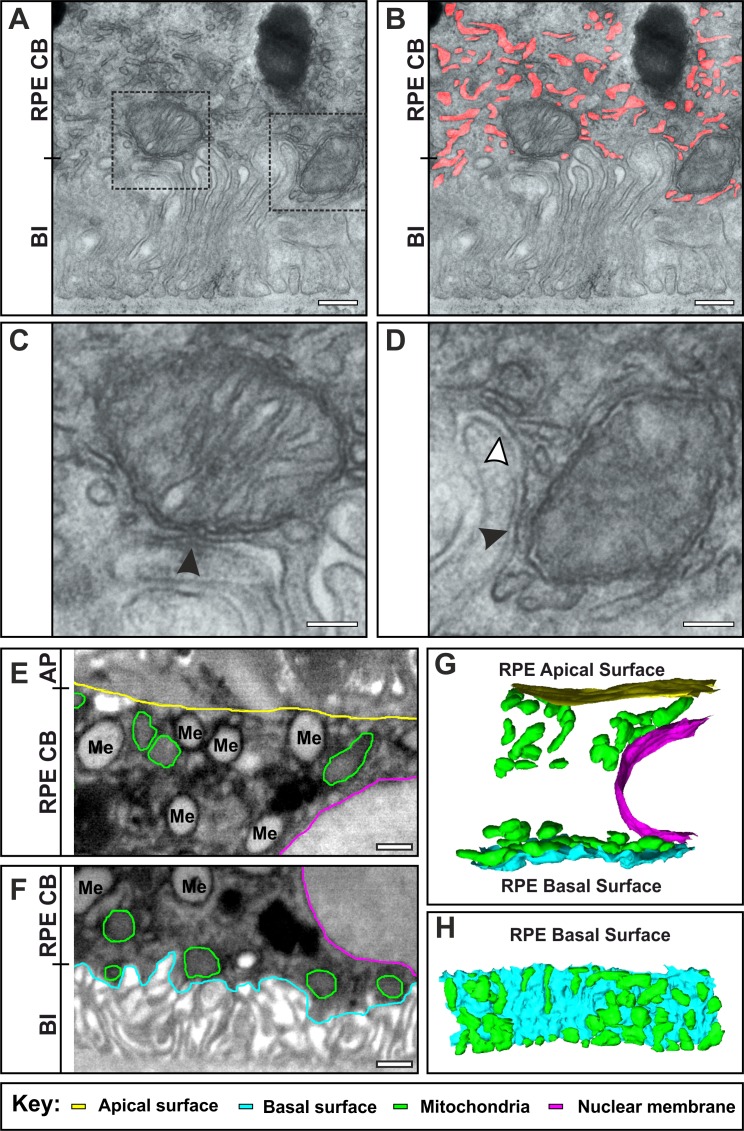
Basal infoldings lack organelles but cisternal elements make membrane contacts with mitochondria and ER. (A–D) Conventional electron microscopy of 5-month-old wild-type mouse RPE reveals a dense network of ER that is highlighted in red in (B), which is absent from the basal infoldings. (C, D) Boxed regions are magnified. Arrowheads indicate contact sites between the basal infolding plasma membrane and mitochondria (black arrowheads) as well as the ER (white arrowheads). (E–H) Single SBF-SEM slices at the apical surface (E) and basal surface (F) of a mouse RPE cell have been outlined in color (apical surface in yellow, basal surface in blue, mitochondria in green, and nuclear membrane in pink) to indicate the structures segmented to form the models shown in (G, H). The longitudinal view shows that mitochondria are more tightly associated with the basal compared with apical surface (G). The en face view shows that mitochondria form a ‘carpet' covering a large proportion of the basal surface (H). Scale bars, (A, B) 250, (C, D) 100, and (E, F) 500 nm.

The physical dimensions of the stacked and ribbon-like zones clearly preclude the possibility of mitochondria being present within them (Fig. 4A) and we found that the endoplasmic reticulum (ER) is also largely absent from these zones (Fig. 4B). However, thin-section TEM clearly shows that mitochondria and the ER make membrane contacts with cisternal elements of the basal infoldings (Figs. 4C, 4D). While mitochondria tend to localize to the basal region of RPE cells (Fig. 4F) they can be present in the apical region but rarely make contacts with the apical plasma membrane (Fig. 4E). Three-dimensional reconstruction of SBF-SEM data emphasizes the close association between mitochondria and the basal cisternal elements in contrast with the much looser association of mitochondria with the nucleus and apical surface (Figs. 4G, 4H).

### Cell Autonomous Shape Changes of the Basal Infoldings in Response to Changes in Osmolarity

**Figure 5:**
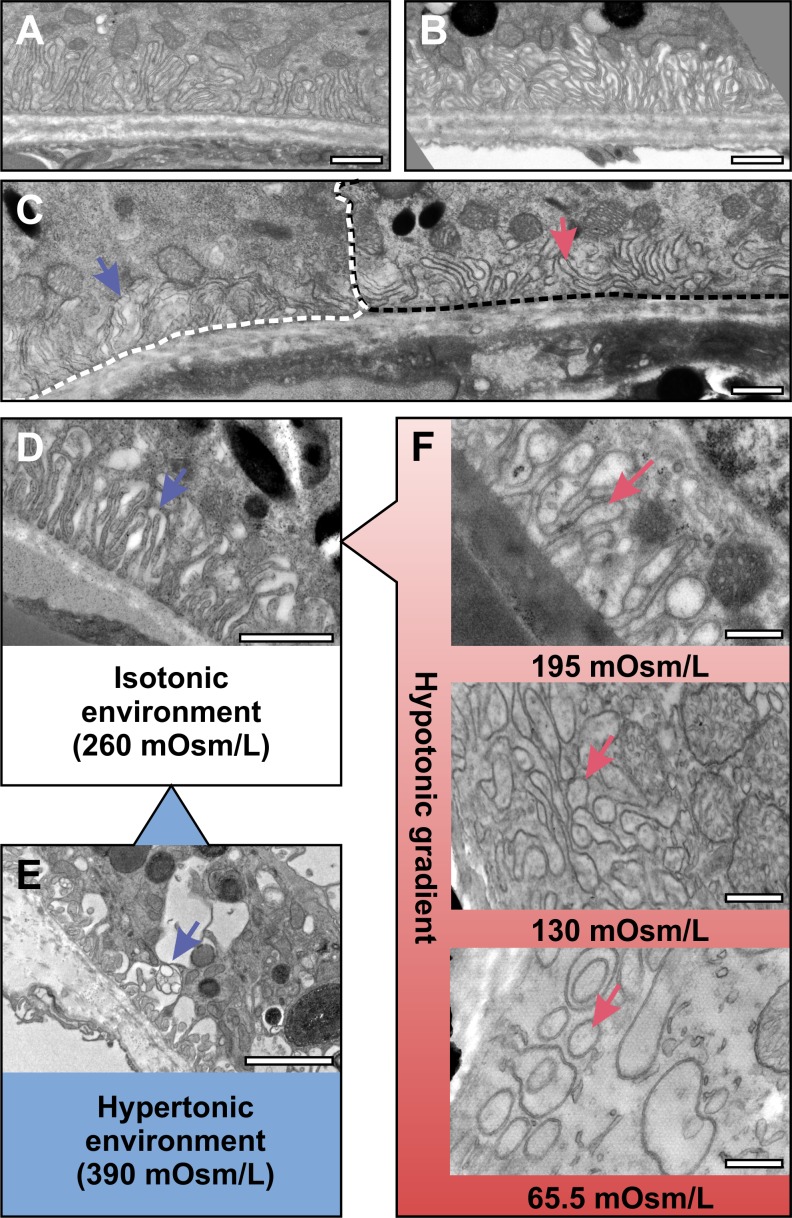
Basal infoldings exist in a ‘open' or ‘closed' state that is influenced by changes in environmental osmolality. (A–C) Conventional electron microscopy of 5-month-old WT mouse eye shows basal infoldings exhibiting an ‘open' state with extracellular space between the plasma membrane of basal infoldings (A) and also tightly associated in a ‘closed' state with no visible extracellular space (B). Neighboring RPE cells can present different basal infolding states (C). The white and black dotted lines highlight the boundaries of two separate RPE cells, and the blue arrow indicates basal infolding in an ‘open', whereas the red arrow in an ‘closed' state. (D–F) Entire mouse eyes were exposed to regular fix (D), hypertonic (E), and hypotonic (F) solutions and examined by conventional TEM. Hypertonic solution causes the extracellular space between basal infoldings to increase while incubation in hypotonic solutions leads to ‘closed' basal infoldings, and extremes of hypotonicity lead to basal infoldings appearing internalized. The blue arrows indicate basal infolding in an ‘open', whereas the red arrow in a ‘closed' state. Scale bars, (A–D) 1, (E) 3, and (F) 1 μm.

The dimensions of the comparatively wide cisternal and progressively narrower paracellular spaces between basal infoldings will influence the nature of the osmotic gradient that forms across the membrane of the infoldings. Interestingly, we noticed that in some specimens the width of the paracellular space was variable such that some regions of the sample had tightly opposed basal infoldings (we termed ‘closed' – Fig. 5A), whereas other areas had clear paracellular space (we termed ‘open' – Fig. 5B). To determine whether these differences were related to chemical fixation we compared the basal infoldings of specimens that had been either conventionally chemically fixed, had undergone the slightly modified chemical processing for SBF-SEM or had been high-pressure frozen (Supplementary Fig. S2). While in SBF-SEM and high-pressure frozen specimens the basal infoldings were largely in the ‘open' state, in some conventionally chemically fixed specimens the space between basal infoldings was reduced to tiny slivers (‘closed'). Although chemical fixation could partially induce the closed state, closed and open cells could exist adjacent to each other (Fig. 5C), suggesting a cell autonomous response to immediate surroundings and not a general response to processing of the entire tissue. The fixation used is slightly hypo-osmotic compared with the likely osmotic milieu of the RPE in situ, suggesting that the transition between ‘open' and ‘closed' state might be regulated by subtle changes in osmolarity.

To examine this further we developed an ‘entire-eye' ex vivo assay for basal infolding structure in which excised eyes were immediately plunged into buffers of varying tonicity, ranging from hypertonic (400 mOsM) to hypotonic (∼65 mOsM) for 10 minutes before fixation and processing for TEM. We identified a number of different basal infolding shapes that were induced by changes in tonicity (Figs. 5D–F). At the extreme end of hypertonicity the stacked membrane was gone, replaced by a limited amount of ribbon-like infolding and very large cisternae (Fig. 5E). At what might be termed ‘expected-physiological osmolality' (the osmolality of the paracellular fluid around the RPE is not precisely known) we saw a transition from an open state with clearly visible paracellular space between the infoldings (Fig. 5D) to a closed state with almost no visible paracellular space (Fig. 5F). This was accompanied by swelling of the basal infoldings and in particular of the filopod-like protrusions, which entered the cisternal spaces. These filled the cisternal space so that the paracellular space was reduced to almost nothing. At extremes of hypo-osmolality the basal infoldings appeared to be fully internalized into the cytosol of the RPE appearing as rings of membrane in two-dimensional sections (Fig. 5F).

## Discussion

We have used SBF-SEM to show that the RPE basal labyrinth is composed of three structurally distinct zones. The geometries of these different zones dictate fundamental properties of the labyrinth, such as the surface area available for contact with Bruch's membrane and cellular organelles and the shape of the osmotic gradient that forms in the paracellular space. Understanding the extent to which the different zones are lost with age and disease will allow the prediction of the likely effects of these changes on blood–retinal barrier function.

**Figure 6:**
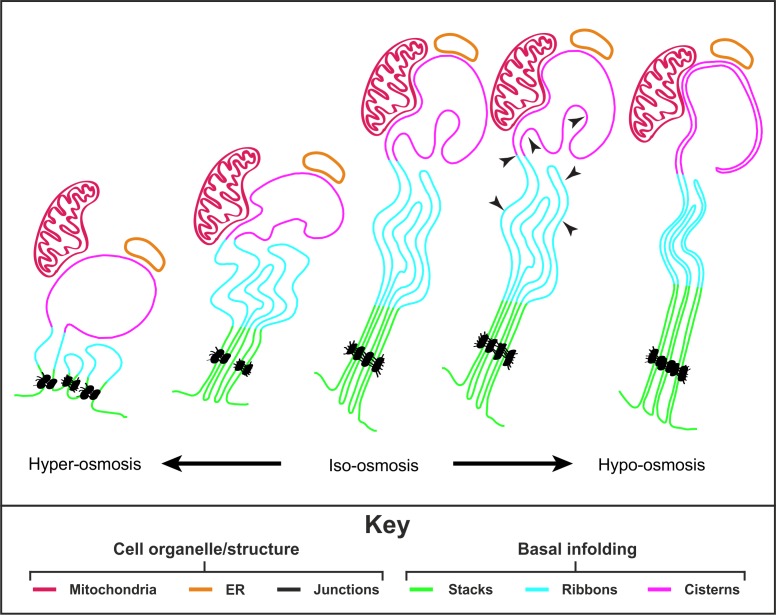
Schematic showing the remodeling of the RPE basal infoldings in response to changes in environmental osmolality. RPE basal infoldings consist of three distinct structural zones, stacks (green), ribbons (blue), and cisterns (pink). Changes in the environmental osmolality result in remodeling of the basal infolding membranes. A hyperosmotic environment leads to an ‘open' state whereas hypo-osmotic results in swelling of the RPE restricting the extracellular space between membranes to give a ‘closed' basal infolding state. Junctions (shown in black) help to maintain the basal infolding structure and mitochondria (red) as well as ER (yellow) are in contact with the plasma membranes of the basal infolding cisterns, likely involved in the process of SOCE. The black arrowheads show the likely direction of intracellular fluid tension on the basal membrane.

In young animals, a significant proportion of the basal membrane is elaborately folded into regular plate-like ‘stacks' with narrow paracellular channels (tubes) between the infoldings (Fig. 6). Adjacent to this highly ordered region is an intermediate zone which has slightly broader cytoplasmic extensions arranged in a more elaborate, less ordered way (Fig. 6). These we have termed ‘ribbons', reflecting the interdigitating extracellular space between them. More ‘apically', next to the bulk of the cell body, the paracellular space opens out into spheroid spaces we term ‘cisterns' (Fig. 6). Into most of these spaces intrude one or more protrusions of evaginated membrane. Only the cisternal region of the basal infoldings makes contact with other organelles, specifically with mitochondria and the ER (Fig. 6).

During normal aging and prematurely in choroideremia, the stacked regions are lost first, followed by the ribbon-like regions, while cisternal elements remain and, in some cases, are increased when the other zones are lost. What underlies this breakdown in structure is unclear. Loss of function of REP1 in CHM causes a hierarchical reduction of Rab prenylation, with Rabs 27a and b, 38 and 42 being the most affected in vitro in a non-RPE model system.^20^ In RPE cells Rab27a and Rab38 regulate melanosome movement and biogenesis respectively^21^–^24^ and basal infolding defects in mouse models lacking Rab27a or Rab38 function have not been reported. The function of Rab42 is unclear and further Rabs may be underprenylated in RPE cells resulting in subtle defects in membrane traffic pathways, the results of which accumulate over time leading to gradual retinal degeneration. Clathrin-dependent and -independent endocytosis and recycling pathways regulate the total surface area of the plasma membrane and the density of ion and water transporters and adhesion molecules and are all potentially affected by defects in Rab protein function. While clearly the plasma membrane surface area available for infolding has the potential to profoundly influence structural organization of the basal labyrinth, interactions between adjacent basal infoldings and between the basal infoldings and the RPE basement membrane will also help to form and stabilize infolding structure. Accumulation of advanced glycation endproduct adducts in Bruch's membrane^25^ and in the extracellular matrix between adjacent infoldings could lead to a loss of recognition by basal infolding adhesion receptors.^26^ Additionally, the age-related accumulation of basal laminar deposits and Drusen may physically modify the structural arrangement of overlying basal infoldings.

The distance between infoldings in stacks is highly consistent and we observed a junctional complex that may maintain the spacing of these membranes (Fig. 6). These junctions were previously described in rat, cat, dog, and human as resembling desmosomes,^27^ which could be either ‘cis-junctions' between basal infoldings on the same cell or ‘trans-junctions' between long-range interdigitating projections of neighboring cells. Subsequent observations in developing chick embryos^28^ and our own observations in the mouse, suggest that they are indeed a novel type of cis-junction that have not generated much attention in the literature, presumably because they are small and best observed in oblique or en face sections. The developing chick RPE apical zonula adherens junction appears by TEM to be generated by aggregation of these junctions, suggesting that their composition may be more similar to that of adherens junctions. We do not know if our failure to identify adherens and desmosome proteins at these sites represents a failure of technique or whether they express atypical components of each or whether they represent a novel form of junction.

The ordered and distinct structural zones within the RPE basal labyrinth suggest that the structure regulates more than simply surface area. Although the relative contributions of paracellular (through the cell–cell junctions) and transcellular water transport remain a subject of debate, at least some water transport is transcellular and so must pass across the basal labyrinth. According to the osmotic coupling theory water transport across the epithelial cell basal membrane can be explained by salt being pumped across the membrane into a ‘space', creating an osmotic difference, and water thus follows by osmosis. Clearly the complex structure of the basal labyrinth generates a space with different geometries in the different structural zones. Diamond and Bossart^29^ explicitly modeled how the space, visualized as a long, thin tube with solutes transported into the closed end, could generate a standing osmotic gradient, the shape of which would depend on the length of the ‘tube', its radius, the water permeability, and the rate and site of solute transport. We have shown that the radius and length of the paracellular space between basal infoldings (the ‘tube') varies from the cisternal to ribbon to stacked zones, becoming progressively narrower and longer (Fig. 6). Furthermore, we have shown that the endoplasmic reticulum and mitochondria are restricted to the cisternal zone, which equates to the closed end of the tube in the Diamond and Bossart model, and so capacitive calcium flux (and the chloride transport that might result from it due to calcium-dependent chloride channels) may be restricted to this region. The short distance (<30 nm) between the outer mitochondrial membrane and the plasma membrane of the cisternal basal infoldings indicate the existence of membrane contact sites.^30^ Interestingly, similar mitochondrial to plasma membrane contact sites have been described in yeast,^31^ but have not been previously characterized by electron microscopy in mammalian cells. These would likely assist in anchoring the mitochondria into position against the plasma membrane, facilitating processes such as store-operate calcium entry (SOCE) from the paracellular space.^32^ Why does the basal labyrinth need to be so complex, rather than a simple array of parallel ‘tubes'? One possibility may be to be able to rapidly change the geometry in order to respond to changes in isotonicity (Fig. 6), which are necessary to maintain RPE cell volume. The variation that we observed in paracellular space between very thin (closed) and wider (open) even within the same specimen, could represent a response to subtle changes in osmolarity. Consistently, we saw major changes in basal labyrinth structure in whole-eye globes experimentally exposed to osmotic challenge (Fig. 6). Loss of the ability to rapidly respond to osmotic changes with age and disease could lead to changes in RPE cell volume eventually leading to RPE cell death. An important area for future study will be to link the changes that we have observed with age and in response to osmotic challenge to functional changes in osmotic pressure and ion transport across the basal labyrinth.

In addition to geometric variation, regional variation in aquaporin and ion channels/transporters may also influence the ionic gradient that forms in the space between infoldings. Localizing aquaporins and ion channels/transporters to the different structural zones will require further refinement of immuno-EM techniques to allow efficient antibody labeling, while maintaining the structural zones of the basal labyrinth in recognizable form.

The complex architecture of the basal infoldings has the capacity to regulate both the interaction of the basal membrane with cytoplasmic organelles and the geometry of the extracellular space and the osmotic gradient that forms within it. Further, impaired basal labyrinth architecture in choroideremia implicates Rab-mediated trafficking in regulating its structure. How defects in Rab protein function and age-related changes in gene expression/posttranslational protein modifications regulate the basal labyrinth architecture will be topics of future study.

## Supplementary Material

Supplement 1

Supplement 2

Supplement 3
