# Supplementary material for: Remodeling of the Basal Labyrinth of Retinal Pigment Epithelial Cells With Osmotic Challenge, Age, and Disease
Source: Invest Ophthalmol Vis Sci. 2019 Jun;60(7):2515–24. doi: 10.1167/iovs.19-26784 (PMC6564051; doi:10.1167/iovs.19-26784)

Supplementary Figure 1. Examples showing changes in the basal infolding structure with age and in the choroideremia (Chm) mouse model. The graphs show the variation of the different basal infolding types in individual *ChmWT* (WT), *Chm^{Flox}* (Flox) and the *Chm^{Flox}Tyr-Cre* (Chm) model mice along the length of the retina. Aging (when comparing 6 month WT against 12 month Flox and 24 month WT) resulted in reduction in stacks, and an increase in flat basal infolding that lacked structure. The 12 month Chm mouse showed a loss of basal infolding stacks and ribbons, indicating a similar trend to the aged, 24 month mouse.

Supplementary Figure 2. Basal infolding architecture following different electron microscopy tissue preparation techniques. When preparing mouse retina tissue for conventional electron microscopy, both 'closed' and 'open' basal infolding states were observed. High-pressure frozen tissue, which provides optimal tissue preservation, and SBF-SEM provided similar quality and morphology of basal infoldings to conventional electron microscopy preparation, but most were in the 'open' state. Scale bars: 500nm.

Supplementary video 1

Serial block-face scanning electron microscopy data of RPE basal infoldings from longitudinally orientated mouse retinal tissue.

Supplementary video 2

Serial block-face scanning electron microscopy data of RPE basal infoldings from en face orientated mouse retinal tissue showing sequential slices starting at the RPE apical surface, through the RPE to the basal infolding and into the Bruch's membrane.

Supplementary Figure 1

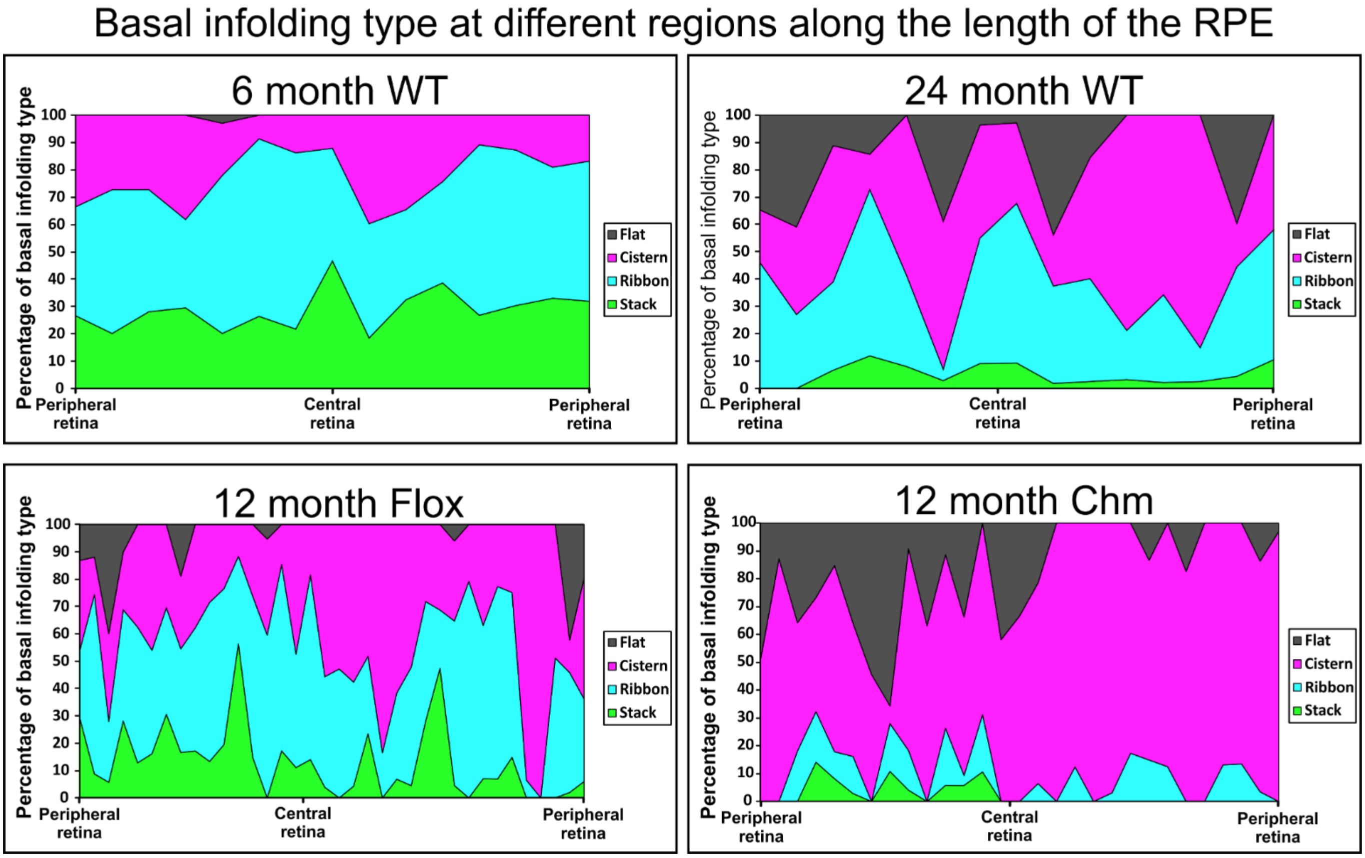

Supplementary Figure 2

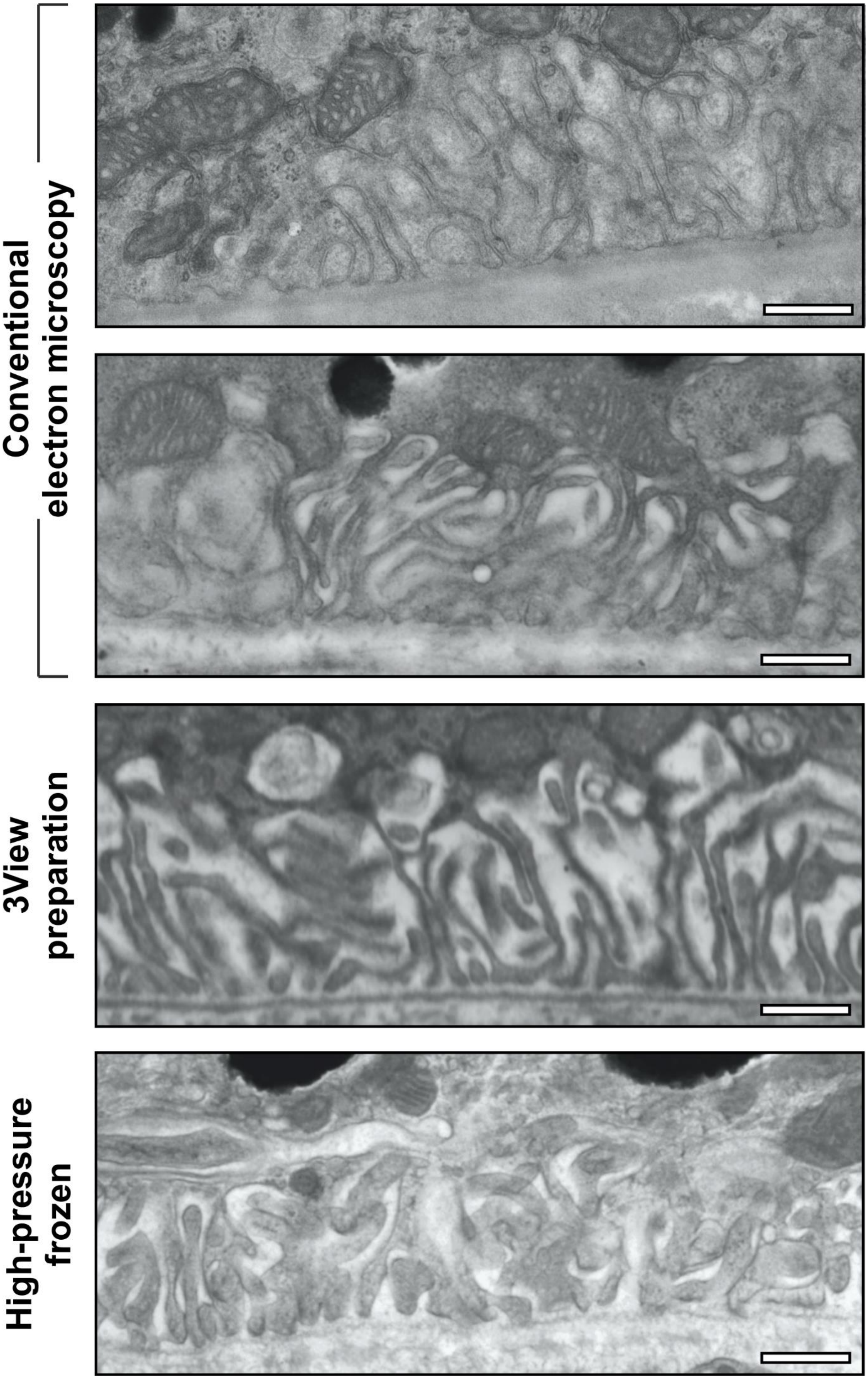

Supplement: Supplement 1 [file iovs-60-07-07_s01.pdf]
